# Scleral thinning surgery for bullous retinal detachment with retinal pigment epithelial tear in central serous chorioretinopathy: a case report

**DOI:** 10.1186/s12886-020-01409-w

**Published:** 2020-04-06

**Authors:** Emilia Maggio, Maurizio Mete, Giorgia Maraone, Fabrizio Arena, Grazia Pertile

**Affiliations:** IRCCS Sacro Cuore Don Calabria Hospital, Via Don Sempreboni 5 - Negrar, 37024 Verona, Italy

**Keywords:** Bullous serous retinal detachment, Chronic central serous chorioretinopathy, Pachychoroid, Retinal pigment epithelial, Sclerectomies, Scleral thinning surgery

## Abstract

**Background:**

Bullous serous retinal detachment (RD) with retinal pigment epithelial (RPE) tear is a rare and severe variant of chronic central serous chorioretinopathy (CSC). Due to its atypical presentation, it may raise diagnostic issues, leading to inappropriate therapeutic procedures. The optimum treatment for this CSC variant is still uncertain.

**Case presentation:**

A 65-year-old male was referred for vitreo-retinal surgery with a provisional diagnosis of rhegmatogenous RD in his right eye. Dilated fundus examination showed an inferior bullous RD with no evidence of retinal breaks, while a large RPE tear was detected in the temporal quadrant. Ocular ultrasound showed no mass lesion. The axial length was 23.63 mm. Enhanced depth imaging optical coherence tomography (EDI-OCT) revealed a pachychoroid pattern in both eyes. The patient referred a history of CSC in the right eye and the recent use of intravenous corticosteroids for bronchitis. Laser therapy and photodynamic therapy were not applicable due to the extension and elevation of the RD. Two months after oral treatment with eplerenone, the subretinal fluid increased significantly. The patient underwent two 4 × 4 mm deep lamellar sclerectomies in the inferior quadrants. The surgical treatment resulted in complete RD resolution.

**Conclusion:**

A correct diagnosis of bullous variant of chronic CSC with RPE tear is critical to avoid inappropriate procedures and to prevent severe visual loss as a result of neuroretinal damage. Scleral thinning surgery may be considered a valid option, resulting in rapid and long-lasting resolution of RD.

## Background

Bullous serous retinal detachment (RD) is a rare and severe manifestation of chronic central serous chorioretinopathy (CSC). It may present with a retinal pigment epithelial (RPE) tear, that, conversely, is not common in eyes with non-bullous CSC. Unlike neovascular age-related macular degeneration, RPE tear in CSC is thought to be the result of tractional forces generated by increased hydrostatic pressure from a fluid-filled pigment epithelial detachment (PED) [1].

The cause for this atypical presentation remains uncertain. It is thought to be an exaggerated form of CSC, resulting from a more pronounced breakdown in the permeability of the choroid and RPE [1–3]. It has not been frequently described as spontaneous; rather, it is mostly associated with a history of corticosteroid therapy, organ transplantation, haemodialysis, or during pregnancy [4–7]. Due to its atypical presentation, the disease may raise diagnostic issues, leading to inappropriate diagnoses of rhegmatogenous RD or serous RD due to other causes. Several previously reported cases [8–10] underwent scleral buckling, vitrectomy, cryopexy, or corticosteroid therapy as a result of misdiagnosis.

However, the optimum treatment for this variant of CSC is still unclear. Effective therapeutic options are limited. Conventional treatments for CSC are often prevented by the extension and elevation of the exudative retinal detachment. Given the hypothesized pathogenetic mechanisms underlying the disease, a scleral thinning surgical treatment, aimed to improve the transscleral outflow, might be considered an effective option. Nevertheless, in the previous literature it has not been undertaken as a viable treatment for the bullous variant of CSC.

Herein, we report the case of a patient affected by the bullous variant of chronic CSC with RPE tear, treated with scleral thinning surgery with a rapid and long-lasting resolution of the disease.

## Case presentation

**Fig. 1:**
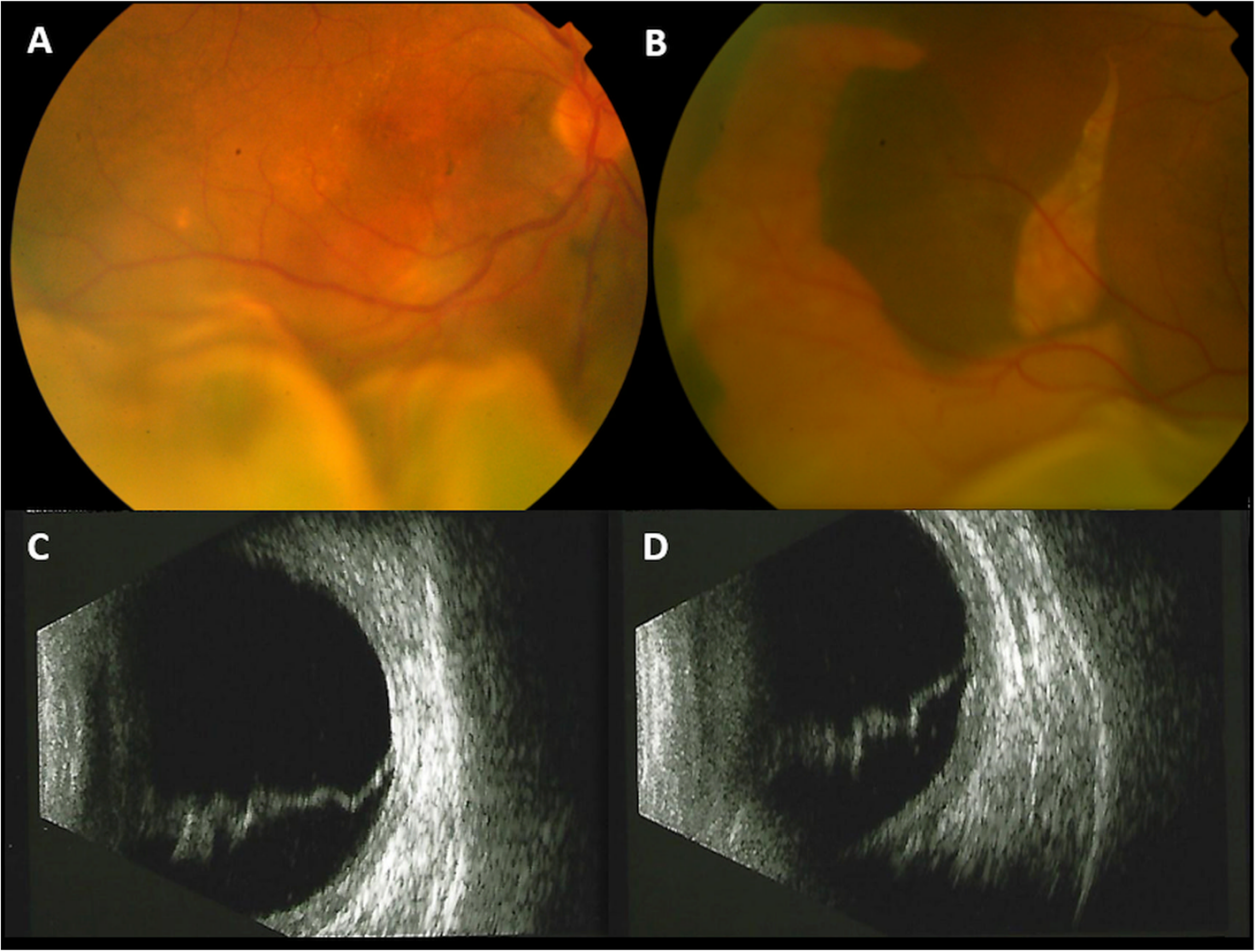
Fundoscopic and ultrasound findings at presentation. **a**, **b**: Color fundus photographs of the right eye showing an inferior bullous retinal detachment involving the macula (**a**) with a large retinal pigment epithelial tear in the temporal quadrant (**b**). **c**, **d**: Ocular ultrasound showing exudative retinal detachment with no mass lesion

**Fig. 2:**
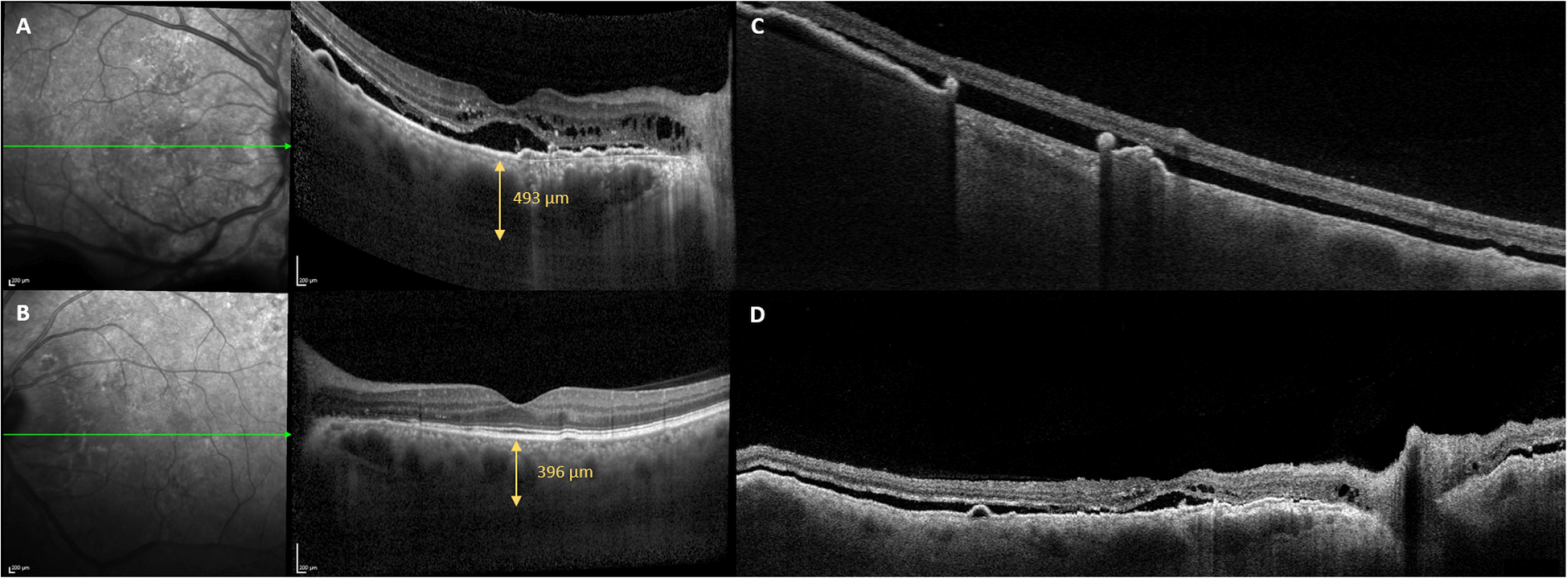
Enhanced depth imaging optical coherence tomography (EDI-OCT) and Swept-Source OCT (SS-OCT-A) angiography findings. **a**, **b**. EDI-OCT revealing a pachychoroid pattern in both eyes (**a**) and sub-retinal fluid reaching the macular area from the inferior quadrants in the right eye (**b**). **c**, **d**. SS-OCT-A showing the mid-pheripheral RPE tear in the inferotemporal quadrant (**c**) and confirming the pachychoroid pattern

**Fig. 3:**
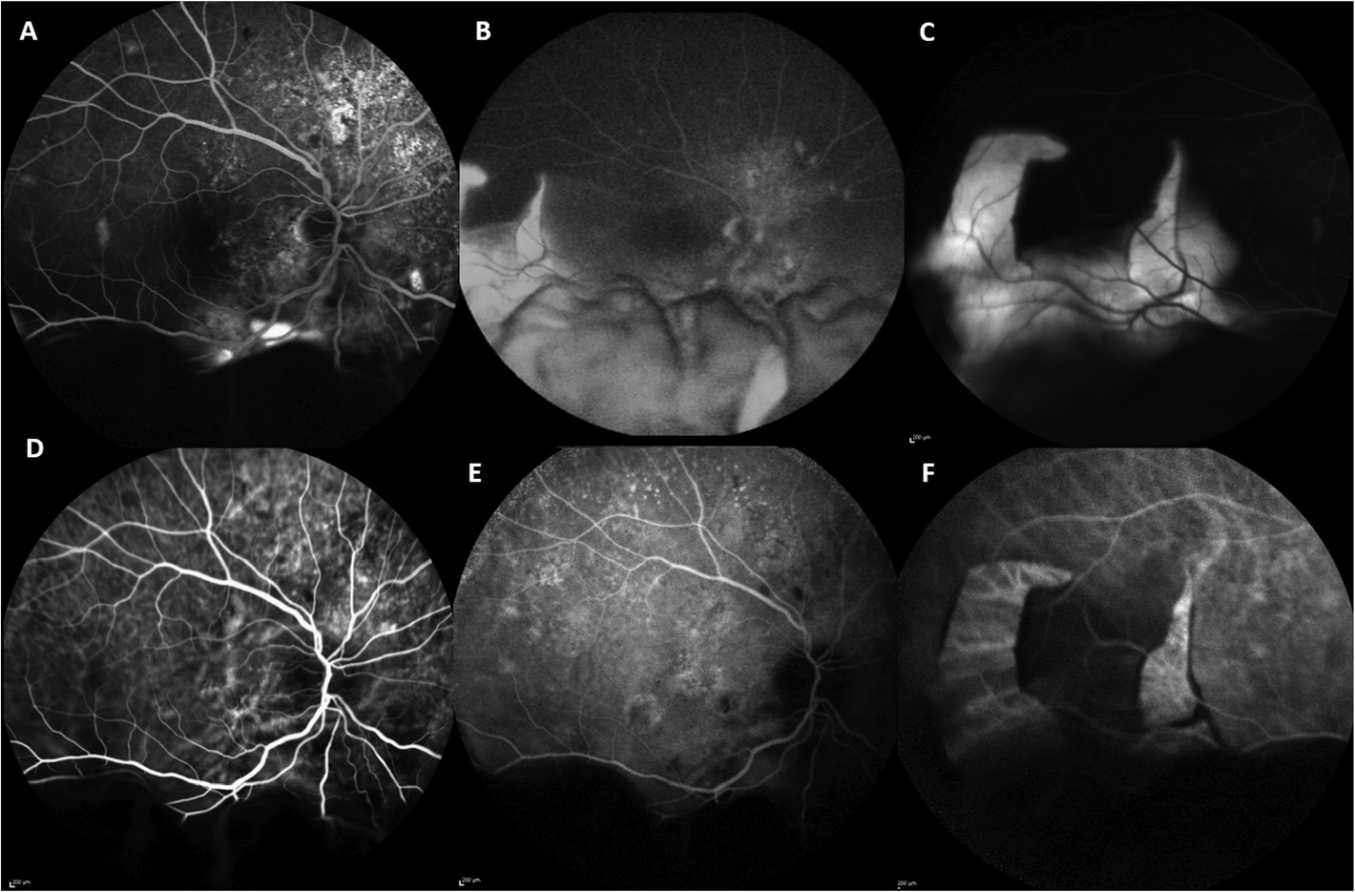
Fluorescein angiography (FA) and Indocyanine green angiography (ICGA) at the presentation, revealing hyperpermeable and dilated choroidal vessels and multifocal and diffuse leakage. **a**, **b**. Early and late FA angiograms. **d**, **e**. Early and late ICGA angiograms. **c**, **f**. FA and ICGA angiograms at the site of the retinal pigment epithelial tear

A 65-year-old white male was referred to our Vitreo-Retinal Surgery Service for a retinal detachment (RD) in his right eye. On examination, his best-corrected visual acuity (BCVA) was 20/63 in the right eye (RE) and 20/20 in the left eye (LE), with a small refractive error. Intraocular pressure was 15 mmHg OU. Slitlamp examination revealed quiet anterior chambers and slight nuclear sclerosis in both eyes. Dilated fundus examination of the right eye showed an inferior bullous RD involving the macula with no evidence of retinal breaks, while a large RPE tear was detected in the temporal quadrant (Fig. 1, A,B). There was no sign of uveitis or vitreitis. Fundus examination of the LE was unremarkable, except for slight RPE distrophic alterations at the posterior pole with RPE mottling. Enhanced depth imaging optical coherence tomography (EDI-OCT) revealed a pachychoroid pattern in both eyes (Fig. 2). Moreover, OCT scans of the right eye showed sub-retinal fluid (SRF) reaching the macular area from the inferior quadrants. No SRF was detected in the LE. Ocular ultrasound showed no mass lesion (Fig. 1, C,D). Upon questioning his medical history, the patient referred a history of central serous chorioretinopathy (CSC) in the RE and the recent use of intravenous corticosteroids for bronchitis. Fluorescein angiography (FA) and Indocyanine green angiography (ICGA) were performed, revealing hyperpermeable and dilated choroidal vessels and multifocal and diffuse leakage in the late angiograms of the RE (Fig. 3). No disc leak or vasculitis were detected. Swept-Source OCT angiography (SS-OCT-A) confirmed the pachychoroid pattern and clearly demonstrated the mid-pheripheral RPE tear in the inferotemporal quadrant (Fig. 2). Axial lengths were 23.63 mm (RE) and 23.35 mm (LE).

**Fig. 4:**
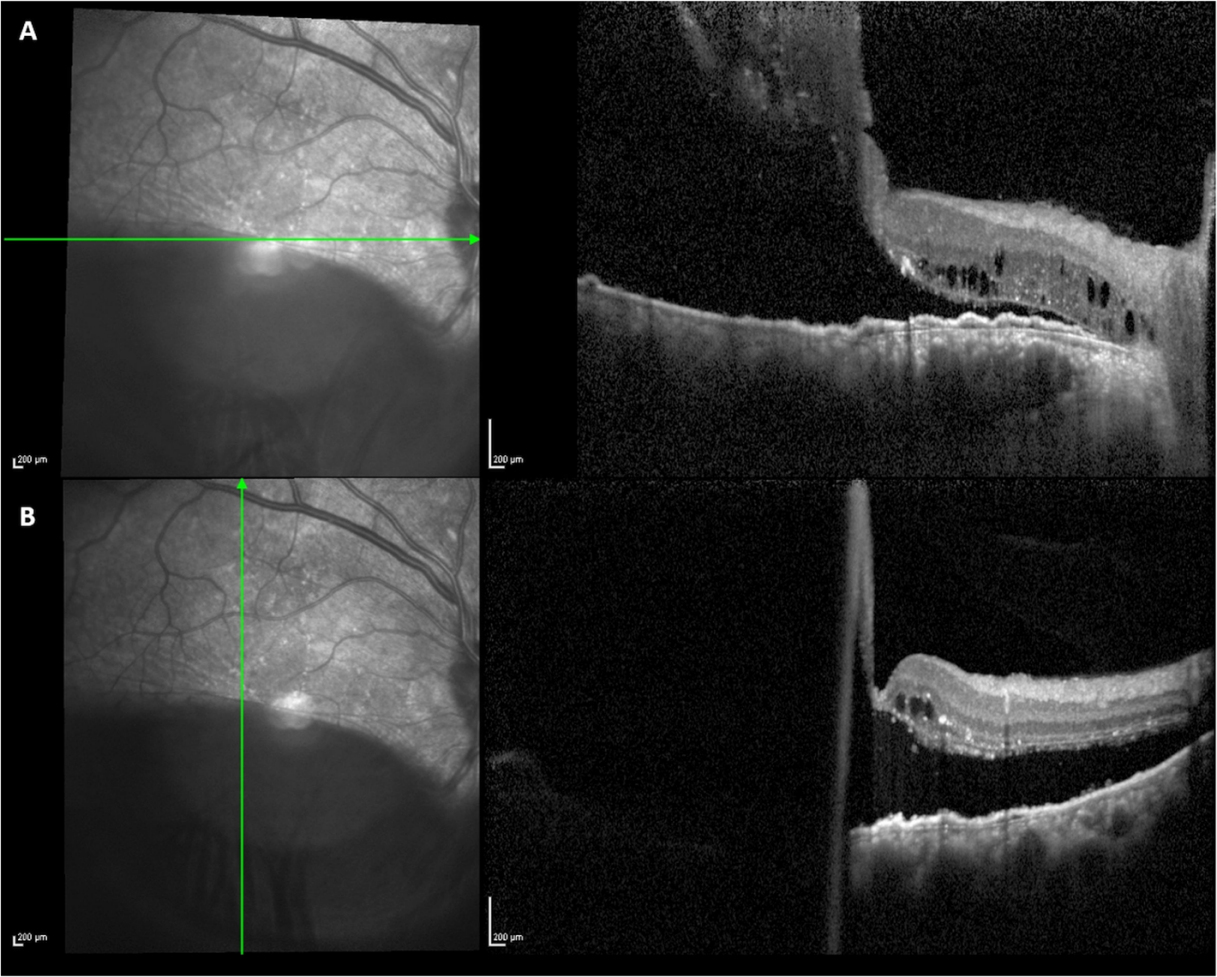
OCT scans performed two months after oral treatment with eplerenone showing the increase in the exudative retinal detachment. **a** Horizontal scan. **b** Vertical scan

Based on the clinical features, a diagnosis of bullous variant of CSC with RPE tear was made. Oral therapy with eplerenone at a dose of 50 mg/day was initiated. However, no improvement was detected. In fact, two months later, the exudative RD increased with a worsening of the BCVA to 20/80 (Fig. 4), despite the continuing therapy with eplerenone. To prevent irreversible photoreceptor damage, management with observation or eplerenone treatment continuation were excluded, and alternative therapeutic procedures were discussed. Laser therapy was deemed inappropriate because the leakage was multifocal and diffuse, with multiple “ink blot” focal areas of leakage at the superior margin of the detached retina and other poorly defined leakage areas in the attached retina. Photodynamic therapy (PDT) was not applicable because of the extension and elevation of the RD. Therefore, the opportunity for surgical treatment was considered.

**Fig. 5:**
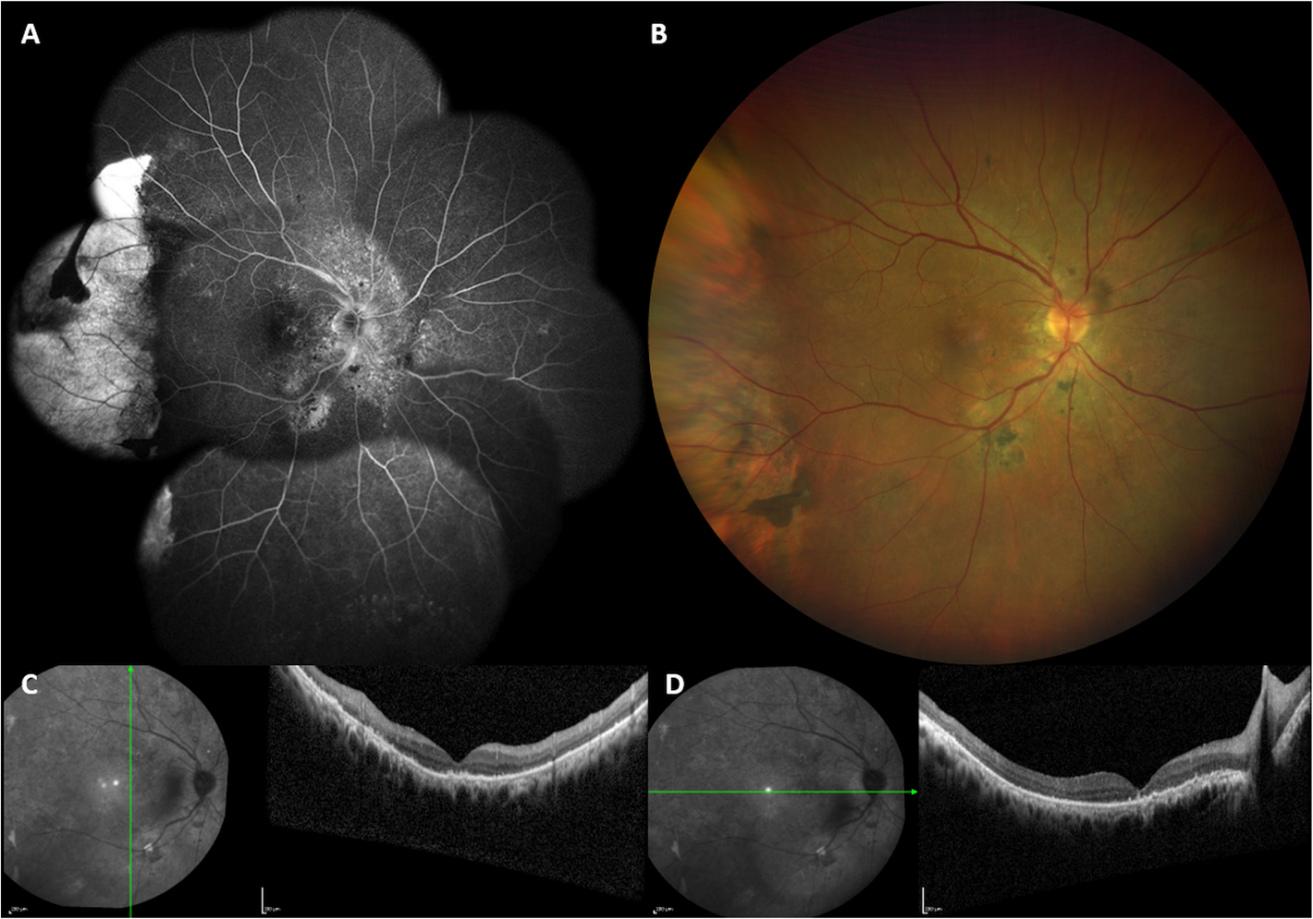
Multimodal imaging performed six months after surgery. **a** Fluorescein angiography showing a sharply demarcated hyperfluorescence (window defect) at the site of the retinal pigment epithelial (RPE) tear and the resolution of the inferior retinal detachment. **b** Color fundus photograph showing the temporal RPE tear and RPE distrophic alterations at the posterior pole. C, D. OCT scans revealing the resolution of subretinal fluid

Written informed consent was given by the patient, after a comprehensive explanation of the procedures. Under general anesthesia, the patient’s eye was prepared with povidone-iodine and draped. After 180° inferior conjunctival peritomy, bridle suture was passed under the inferior, medial and lateral rectus muscles with 3–0 ticron, and the sclera was exposed. Two 4 × 4 mm almost full scleral thickness sclerectomies were done in the inferior quadrants, taking care to avoid the areas close to exit sites of the vortex veins. Finally, the conjunctiva was approximated with 8–0 Vicryl suture. The treatment resulted in complete RD resolution as of the first day after surgery. Upon follow-up six months after surgery- complete SRF resolution was confirmed and BCVA was stable at 20/80 (Fig. 5).

## Discussion and conclusions

Bullous serous RD is a rare complication of chronic CSC. Since the original description by Gass in 1973 [11], there have been only a limited number of case series and case reports that have described the clinical features and management of this CSC variant [1, 12–14].

We report the case of a patient referred to our hospital for vitreo-retinal surgery with a provisional diagnosis of rhegmatogenous RD. However, upon examination, the clinical presentation was not consistent with rhegmatogenous RD, nor with inflammatory exudative RD. Causes of exudative RD include hypertension, VogtKoyanagi-Harada syndrome, metastasis or other intraocular tumors, posterior scleritis, uveal effusion syndrome (UES), and nanophthalmos. In our patient, blood pressure was normal and there was no evidence of intraocular inflammation or posterior scleritis. Mass lesions were excluded by ocular ultrasound. Nanophthalmos and UES were ruled out as the axial length was normal and there was no ciliochoroidal detachment. Bilateral increased choroidal thickness, recent use of intravenous corticosteroids, RPE tear, and RPE dystrophic changes led towards a diagnosis of bullous variant of CSC.

Current treatment options for chronic CSC include laser therapy, PDT and oral mineralocorticoid receptor antagonists.

In patients with the bullous variant of CSC, previous authors have found no significant difference between laser treated and non-laser treated eyes with regards to SRF resolution and final visual outcome [3]. This suggests laser treatment is unable to change the natural course of this disease. Moreover, for our patient, the laser was deemed inappropriate because the leakage was multifocal and diffuse.

PDT has been employed extensively for the treatment of chronic CSC. However, its efficacy for the bullous variant with retinal tears has not been established. Ng et al. [12] described SRF resolution after three months in a case of bullous RD secondary to chronic CSC treated with PDT with half-dose verteporfin. However, the detachment was much less pronounced when compared with our case, and no RPE tear was present. In our case, the elevation of the retina was greater, and the RD was even more extensive after two months of FU following the eprelenone treatment. Therefore, PDT was not applicable.

Oral mineralocorticoid receptor antagonists have been described to induce a decrease in or resolution of SRF in CSC cases that do not spontaneously resolve. Moreover, a previous report describes a case of a bullous CSC variant successfully treated with spironolactone [13]. However, in our case, treatment with oral eplerenone provided no benefit since SRF increased despite the treatment.

Previous authors have also described a case of bullous variant of chronic CSC in an eye with borderline-low axial length, successfully treated with partial thickness scleral resection with mitomycin C, suggesting the improved transscleral outflow as the mechanism underlying the procedure’s efficacy [14]. The authors hypothesized that in smaller eyes, there may be a component of reduced transscleral outflow similar to UES that, in the presence of other coexisting pathologies like chronic CSC, makes it unable to balance the marked exudative inflow. Therefore, they suggested scleral resection surgery for the bullous variant of chronic CSC in small axial length eyes.

Scleral resection surgery has been successfully employed to treat exudative retinal and ciliochoroidal detachment in UES. In those cases, the pathogenesis is thought to be related to scleral abnormalities with alterations of the transscleral diffusion of choroidal extravascular proteins and the accumulation of fluid in the choroid as a result of increased osmolarity [15, 16]. In eyes affected by UES, scleral thinning procedures have been shown to enhance transscleral protein diffusion, thus leading to the resolution of ciliochoroidal and serous retinal detachment [17].

To the best of our knowledge, no previous reports describe the management of the bullous variant of CSC in normal axial length eyes with scleral thinning surgery. Although the pathogenesis of the bullous variant of CSC is partially understood, evidence suggests that it is related to a more pronounced congestion and breakdown in the permeability of the choroidal vessels and RPE [1]. Therefore, it can be hypothesized that an improvement in transscleral outflow would be beneficial for these eyes, also in the presence of normal axial length. The rapid and long-lasting resolution of the bullous RD in our patient confirmed the efficacy of this treatment.

In previous literature, managing the bullous variant of CSC with alternative surgical procedures, such as scleral buckling, vitrectomy, cryopexy or internal drainage, has been described mostly as a result of misdiagnosis [8]. The scleral thinning surgery, when compared to vitrectomy with internal drainage, has the advantage of being a less invasive procedure with a reduced risk of complications, and producing a long-lasting improvement in transcleral outflow. Considering the chronicity of the disease, this might be additionally beneficial for long-term follow-up in these eyes.

The present case suggests that scleral thinning surgery may be considered a valid option for the treatment of bullous variant of chronic CSC with RPE tear, above all when alternative available therapeutic options, such as PDT and laser therapy, are not applicable.

## Data Availability

All data and material are included in the manuscript and the figures.

## Abbreviations

CSC: Central serous chorioretinopathy
BCVA: Best-corrected visual acuity
EDI-OCT: Enhanced depth imaging optical coherence tomography
FA: Fluorescein angiography
FU: Follow-up
ICGA: Indocyanine green angiography
LE: Left eye
PDT: Photodynamic therapy
PED: Pigment epithelial detachment
RD: Retinal detachment
RE: Right eye
RPE: Retinal pigment epithelial
SRF: sub-Retinal fluid
SS-OCT-A: Swept-Source OCT angiography
UES: Uveal effusion syndrome
